# Image and attribute dataset for 16 464 post-consumer garments discarded or donated in Norway

**DOI:** 10.1016/j.dib.2026.113100

**Published:** 2026-07-23

**Authors:** Johan Berg Pettersen, Solveig Aarak, Edyy Vanessa Peña Benítez, Nazia Nourin Moury

**Affiliations:** Industrial Ecology Programme, Department of Energy and Process Engineering. Norwegian University of Science and Technology, Norway

**Keywords:** Apparel, Clothes, Recycling, Second-hand, Extended producer responsibility, Circular economy, Sorting

## Abstract

There is an increasing focus on the sustainability of clothes and textile consumption. European policymakers seek to increase resale and recovery of textiles, as recycled fiber material represents only a very minor proportion of total textiles production. Data for product and material composition and other qualities is in demand.

The database presents image and attribute data for post-consumer clothes. Sample size is 16 464 garment items, equivalent to 4 157 kg of post-consumer clothes collected in 6 urban regions of Norway. Samples are sorted from households to “clothes, shoes and other textile products sorted for reuse” (Donation), and “dry and clean clothes and textile not for reuse” (Textile waste), collected at central recycling stations and distributed donation boxes. Samples undergo visual inspection of garment and product care label, and individual garment photography, to produce (1) a data table of garment attributes, and (2) a dataset of images. Sample attributes include product category; weight; a five-level score for degree-of-wear; brand; intended user; manufacturing method; number of layers; several other attributes that are numerical (velcro, metal and plastic buttons, metal and plastic zippers, metal rivets/eyelets/aglets, pearls, reflectors, and holes front and total) or Boolean (sequins, print, print >100 cm2, all-over print, other product parts, care label presence and readability, home-made, price tag, waterproof membrane). Care label information is used to record year and country of production, and fiber blend for individual layers of the garment.

The data has multiple potential uses, among them the estimation of fiber blends, fiber recovery potential, resale potential and sorting for resale routines, and product lifetime estimation. Generally, the information can inspire and inform innovation in circular economy for textiles, inform extended producer responsibility (EPR) actors and direct sustainable textile policy.

Specifications Table

**Table:** 

Subject	Engineering & Materials science
Specific subject area	Textile Waste Composition and Management
Type of data	Table, Images
Data collection	Numeric and Boolean attributes and images of post-consumer garment samples from observation of samples and their care labels in June-August 2025. Samples are collected from six urban areas of Norway, from central and distributed donation boxes sorted as “clothes and textiles sorted for reuse” or “dry and clean clothes and textile not for reuse”. Data cleaned for uniformity and quality to generate a final sample set of 16 464 garments. (1) Norway post-consumer garment index and attributes: data table. (2) Norway post-consumer garment images: photographic image dataset.
Data source location	Datasets were collected in partnership with public and private collectors in Norway: Oslo, Trondheim, Stavanger, Kristiansand, Sandnes and Ålesund. Data is stored in Zenodo as: NPCGA_datatable_1.1.csv (data table); README_for_datatable.pdf (data table explanation); and images in 6 zip archives NPCGI_Axxxx (Ålesund), NPCGI_Bxxxx (Oslo), NPCGI_Cxxxx (Stavanger), NPCGI_Dxxxx (Sandnes), NPCGI_Exxxx (Kristiansand), NPCGI_Fxxxx (Trondheim).
Data accessibility	Repository name: Zenodo Data identification number (doi): 10.5281/zenodo.18821900. Direct URL to data: https://doi.org/10.5281/zenodo.20440761
Related research article	Not applicable.

## 1 Value of the Data

The data widens the empirical background to discuss potentials for, and limits of, recovery of product and material value in post-consumer garments in a European setting. The following paragraphs describe in a broad sense some relevant applications of the data.

• The width and depth of data guides development of sampling protocols for garment streams for use in further research in textile waste and by waste management operators.
• Fiber blends, presence of disruptors and their representation in garment types and degree-of-wear score informs fiber recovery strategies and need for pre-sorting and pre-processing in textile fiber recycling. This is highly useful for companies seeking to recycle fiber, and actors in extended producer responsibility (EPR) ecosystems.
• Degree-of-wear for garments post-consumer describes suitability for reuse and resale and informs textile and other value-chain actors to consider and develop eco-design and circular business development, such as design thinking for lease or rent of clothes.
• Production year and country informs policies for lifetime extension, decision support to assess environmental impacts of current or alternative life cycle designs with Life Cycle Assessment (LCA), and material efficiency strategies in product and material recovery systems with Material Flow Analysis (MFA). Production year allows analysis of lifetime as a function of garment type, fiber blend and brand.
• Composition of post-consumer streams and how they are sorted for donation or discard is useful to understand sorting behavior. The data allows a starting reference for longitudinal analysis in Norway, and guide improved sample collection in research and waste management planning.
• Combination of Boolean and numerical attributes and brand name with photographic images provides a data rich sample set to develop machine learning routines and link data with other online information, develop strategies and incentives for EPR, and potentially verify the role of fast fashion as driver for textile consumption.

## 2 Background

A substantial share of discarded household textiles is technically suitable for reuse or can be used for material recovery [1,2]. Garment and fiber composition is routinely estimated from put-on-market statistics [3,4], considering few product-specific attributes and primarily aggregated to fiber blends. Only a few studies report garment-level composition or structural details relevant for recycling, such as trims, fasteners, or multilayer constructions on garment level; see e.g. [5,2], or aggregated to entire flows [6]. Image-based datasets primarily describe image-to-fiber relations; see e.g. [7,8]. With few exceptions, e.g., [9,5], large garment datasets rely on near-infrared fiber spectroscopy, e.g., for recognition of fiber material [6,10,11] or weave [12].

Requirements for separate textile collection and extended producer responsibility are increasing, placing demands on more granular and data-rich efforts to characterize and assess the resource potential and constraints for reuse and material recovery from discarded and donated textiles.

## 3 Data Description

The data consists of 16 464 garment samples documented by one csv data table and image files in six libraries. The csv data file contains 79 columns covering multiple garment properties.

The final data table is stored in the NPCGA_datatable_1.1.csv file. Every item is documented by at least one photographic image, identifiable in one of the 6 image zip archives.

Only a very small number of samples in the data lack a photograph.

## 4 Experimental Design, Materials and Methods

### 4.1 Workflow

**Fig. 1:**
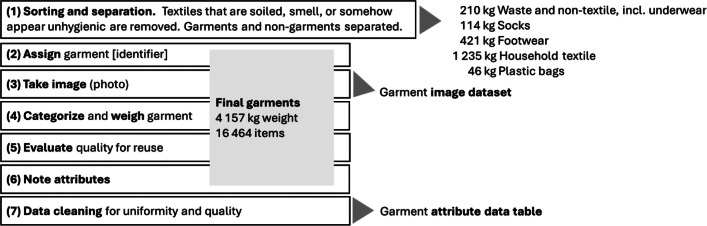
Workflow and data generation steps.

The textiles are sourced from six collectors in Norway, from collection points at central recycling facilities, and donation boxes distributed in residential areas. The workflow is outlined in Fig. 1. From 6 183 kg post-consumer textiles, 2 026 kg of waste and non-garment products were removed, before items were weighed, photographed, categorized, and evaluated for degree -of-wear and product attributes. Proportions of socks, footwear, household textiles, plastic bags, and waste and non-textile including underwear in the streams is detailed per area in Supplementary Information Table 1. The final dataset describes 4 157 kg of clothes, or 16 464 individual garments.

Data cleaning was done to remove incomplete data points and standardize naming and attribute classification. Incomplete data points were considered registrations missing either identifier, garment classification, weight, color, or where they contained obvious errors. Where possible, image files were used to resolve missing registration.

### 4.2 Textile samples

Table 1 describes the samples, sources from either “donations of clothes, shoes and textile for reuse” (Donation), or “dry and clean textile waste not for reuse” (Textile waste). Garments were collected through central sorting containers at recycling stations, or via network of distributed donation boxes. Soiled and wet textile should be sorted from households to the “residual waste” fraction and not appear in either of the two flows. Garments sourced from textile waste represent 1 650 kg (6 576 items) of garments samples, while donations for reuse make up 2 507 kg (9 888 items).

**Table 1:** Sources and amounts of samples. Distributed collection is collection through distributed collection boxes; Central collection is collection at central recycling stations. T. waste denotes the stream of “Textile waste not for reuse”.

Area	Collector, Site	Collection	Sorted as	Mass (kg)	Items (-)	Note
Ålesund	Attvin, multiple	Central	T. waste	815	3 158	(1)
Trondheim	TRV, Heggstadmoen	Central	T. waste	202	714	(2)
Trondheim	TRV, Heggstadmoen	Central	Donation	379	1 426	(2)
Stavanger	UFF Norge boxes	Distributed	Donation	697	2 916	(3)
Sandnes	IVAR, Forus	Central	T. waste	634	2 704	(4)
Kristiansand	Avfall Sør, Mjåvann	Central	Donation	738	2 890	(5)
Oslo	UFF Norge boxes	Distributed	Donation	698	2 656	(6)

(1) Collected from several central locations within greater Ålesund area.

(2) Donation and textile waste containers side-by-side at the Heggstadmoen central facility.

(3) Donation boxes in Stavanger area: Bryne/Klepp, Tananger, Kopervik/Karmøy.

(4) Separate containers for “Textile waste not for reuse” next to donation boxes at the Forus site.

(5) UFF Norge and Blå Kors donation boxes side by side at the Mjåvann site.

(6) Donation boxes in Oslo area.

### 4.3 Textile attributes

Garment categories and sample size in the dataset are described in Table 2. Categories follow Nørup et al. [2] with some exceptions, as separate categories were defined to describe thicker upper-part garments (Sweater, cardigan and other pullover to wear indoor: category 26), headwear (category 27), and thermal underwear in wool or synthetics (category 28). Other underwear, socks, parts and pieces of clothes were removed prior to categorization as outlined in Fig. 1.

**Table 2:** Garment categories and sample size. Category 1–25 numbered according to Nørup et al. [2], with new categories 26–28.

Garment category	Mass (kg)	Items (-)	Category note
1. T-shirts	370.9	2 536	
2. Tops	111.0	923	
3. Blouses	40.0	253	
4. Shirts	156.0	720	
5. Trousers	1 054.0	3 070	
6. Shorts	129.8	644	
7. Winter clothing	83.2	135	
8. Dresses/jumpsuits	216.4	804	
9. Skirts	47.9	227	
10. Vests	26.0	133	
11. Jackets	552.1	914	
12. Infants’ clothes. incl. socks & gloves for babies	36.4	496	
13. Workwear	125.9	185	
14. Apron	4.0	20	
15. Swimwear	18.9	222	
16. Underwear	na.	na.	Not used
17. Nightwear	18.7	112	
18. Bathrobes	22.3	37	
19. Socks	na.	na.	Not used
20. Gloves & mittens (singles)	17.0	437	
21. Scarfs, ties & belts	50.8	579	
22. Handkerchiefs	0.9	47	
23. Costumes	11.0	52	
24. Parts of clothing	na.	na.	Not used
25. Pieces of clothing	na.	na.	Not used
26. Sweater, cardigan and other pullover to wear indoor	970.7	2 957	New
27. Headwear, hats etc.	43.2	610	New
28. Underwear, thermal type	54.7	351	New
Total	4 161.7	16 464	

**Fig. 2:**
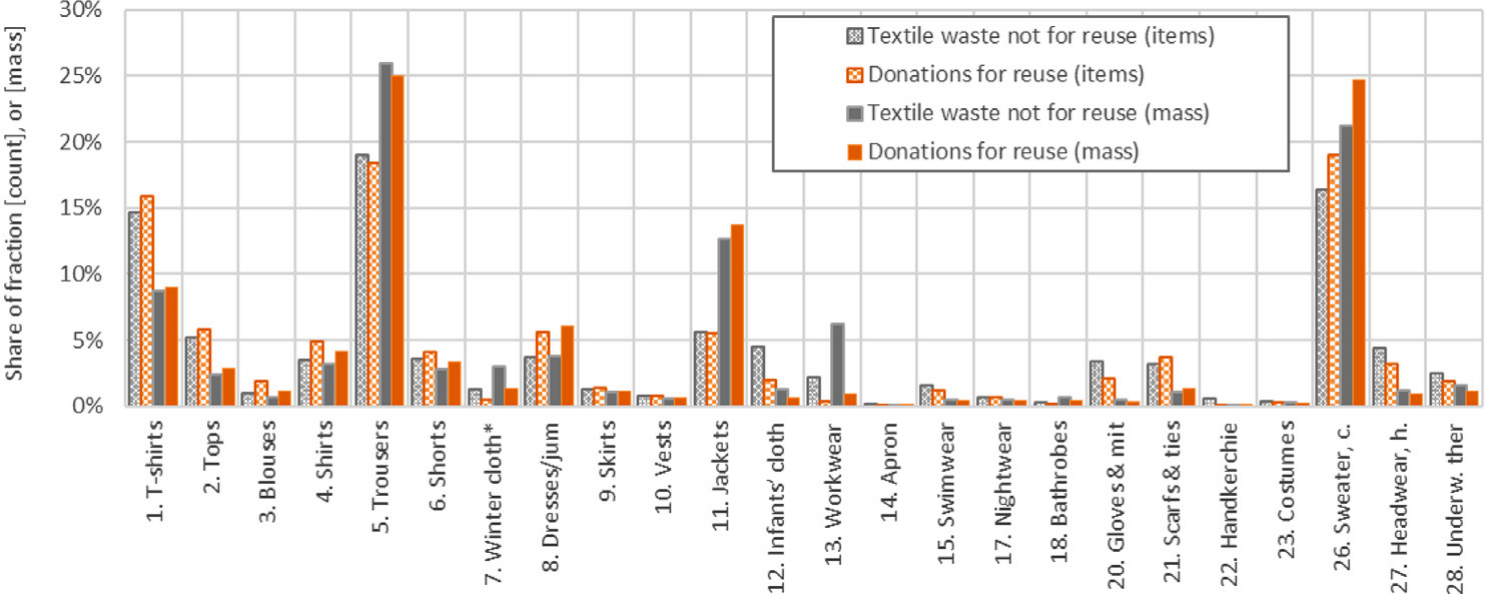
Distribution of garment categories by share of mass or items in Donations for reuse and Textile waste not for reuse. Complete category names provided in Table 2.

Product category distribution is presented in Fig. 2, as total mass and sample numbers in donations for reuse and textile waste not for reuse. The share of garment categories is fairly similar between the two streams, with the main exception being a higher share of workwear in textiles sorted as waste.

Each individual garment is indexed toward attributes in the data table and the image dataset. A small number of garments miss photographic image. The complete attribute list is outlined in Table 3, with Boolean and numeric attributes further detailed in Table 4.

**Table 3:** Dataset elements attributes with * (star) are made by subjective evaluation.

Element of the data table	Description
**Index (Axxxx)**	Individual garment index in Data table and Image dataset. First letter refers to source area; A-F is Ålesund, Oslo, Stavanger, Sandnes, Kristiansand, Trondheim, respectively.
**Area**	6 urban areas; see Table 1.
**Collector**	Collector in each area; see Table 1.
**Sorted as**	Donation for reuse, or Textile waste not for reuse; see Table 1.
**Collection**	Central recycling facility, or distributed donation boxes; see Table 1.
**Product type**	Garment category; see Table 2**.**
**Weight**	Grams
**Color**	Black, Blue, Brown, Green, Grey, Orange, Pink, Purple, Red, White, Yellow, Multi-color, Multi-color pattern, Multi-color stripes.
*Manufacturing method	Knitted (k), woven/tricot (t), other (o), unknown (u).
*Wearer/purpose	Female (f), male (m), unisex (u).
*Age category	Adult (a), child (c), baby (b).
**Brand source**	Brand identifiable in care label: yes (1), no (0).
**Brand name**	From the care label
**Country of production**	From the care label; reported in ISO 3166-1 Alpha-3 codes
**Year of production**	From the care label
*Degree-of-wear	Graded scale for condition and degree-of-wear; see **explanation in text.**
**Number of layers**	From visual inspection
**Fiber blend**	Care label information is used to specify fiber composition for individual garment layers; see **explanation in text.**
**Boolean attributes**	10 attributes for presence or state (Boolean “yes/no”); see Table 4.
**Numeric attributes**	10 numeric attributes (“count of”); see Table 4**.**

**Table 4:** Numeric (count of) and Boolean (yes/no) attributes. Attributes with * (star) are made by subjective evaluation.

Numeric attributes, count of	Boolean attributes, yes (1) or no (0)
Velcro	Contains sequins
Metal buttons	Contains print
Plastic buttons	Contains print > 100 cm²
Metal zippers	Is all-over print
Plastic zippers	Contains other extra parts
Metal rivets, eyelets or aglets	Care label is present
Pearls	*Care label is readable
Reflectors	*Garment is home-made
Holes in front	Price-tag is attached
Holes total	Has waterproof membrane

Some data points are sensitive to subjectivity. The technical state of a garment is measured on a scale of 1 to 5 according to the protocol outlined by Sunde et al. [13] by subjective evaluation. Other records made by subjective evaluation include the intended gender and age category of a garment, as well as manufacturing method and whether the garment is home-made. Attributes concluded by subjective evaluation are marked with * (star) in Table 3, Table 4.

To avoid empty cells, we use NaN (Not a Number) in the data table when information is missing or not identifiable. The following reclassification edits were made for non-standard countries; Czechoslovakia: CSK; West Germany: DEU. Wales, Scotland, England, Britain: GBR. *Europe* and *Scandinavia* lack country codes in ISO 3166-1 and were coded EUR and SKV, respectively.

Several of the attributes are evaluated subjectively, including degree-of-wear, intended user, age category, manufacturing method, home-made classification, as indicated in Table 3, Table 4. The individual criteria were discussed with the sampling team, and for the degree-of-wear supported by image guide and description by Sunde [13]. However, no inter-rater reliability assessment, observer agreement analysis, or calibration exercise was performed.

### 4.4 Degree-of-wear

**Fig. 3:**
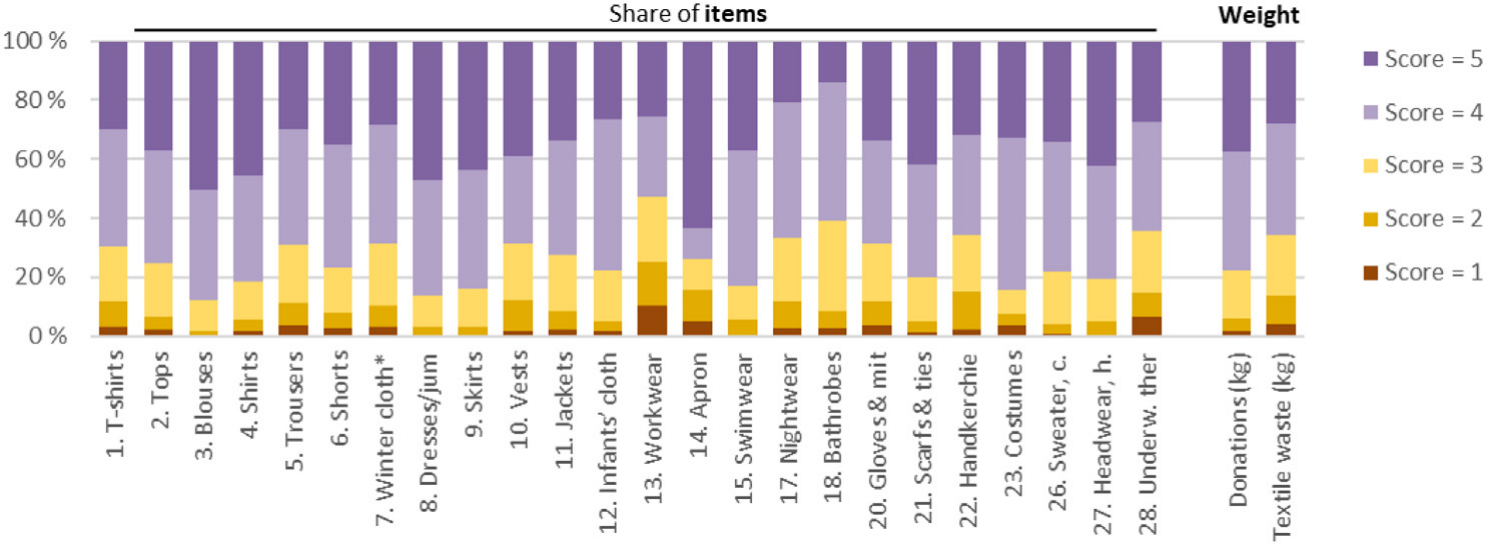
Distribution of degree-of-wear by share of items for garment category, and by share of mass for Donations for reuse and Textile waste not for reuse (right-most columns). Garments score 5 (no noticeable wear) and 4 (minor wear) are well suited for reuse, garments scored 1–3 require significant repair or are generally not fit for reuse. Complete category names provided in Table 2.

Garment condition and degree-of-wear is graded on a scale from 1 to 5 and intended to represent suitability for reuse and resale. The scale is described in detail elsewhere [13], with image guidelines to separate a garment that is not usable and cannot be repaired (1), or not usable but can be repaired (2), or show noticeable change from use (3), or show minor change from use (4), or a garment that in perfect condition and appears as new (5). This follows principally the structure applied in prior study in the Nordics [2]. Fig. 3 shows the degree-of-wear for shares of garment items per category, and as share of total mass of samples in donations for reuse and textile waste not for reuse.

### 4.5 Layers and fiber material

Fiber material is classified according to the EU nomenclature [14]. “Feather or down”, “bamboo” and “leather” are added to the list. We state “other” were such is stated on the label, and NaN when fiber content in layer 1 is unclear. Fiber share is stated for each layer of the garment, as identified in the care label, with layer 1 representing the dominant layer in multi-layer garments.

**Fig. 4:**
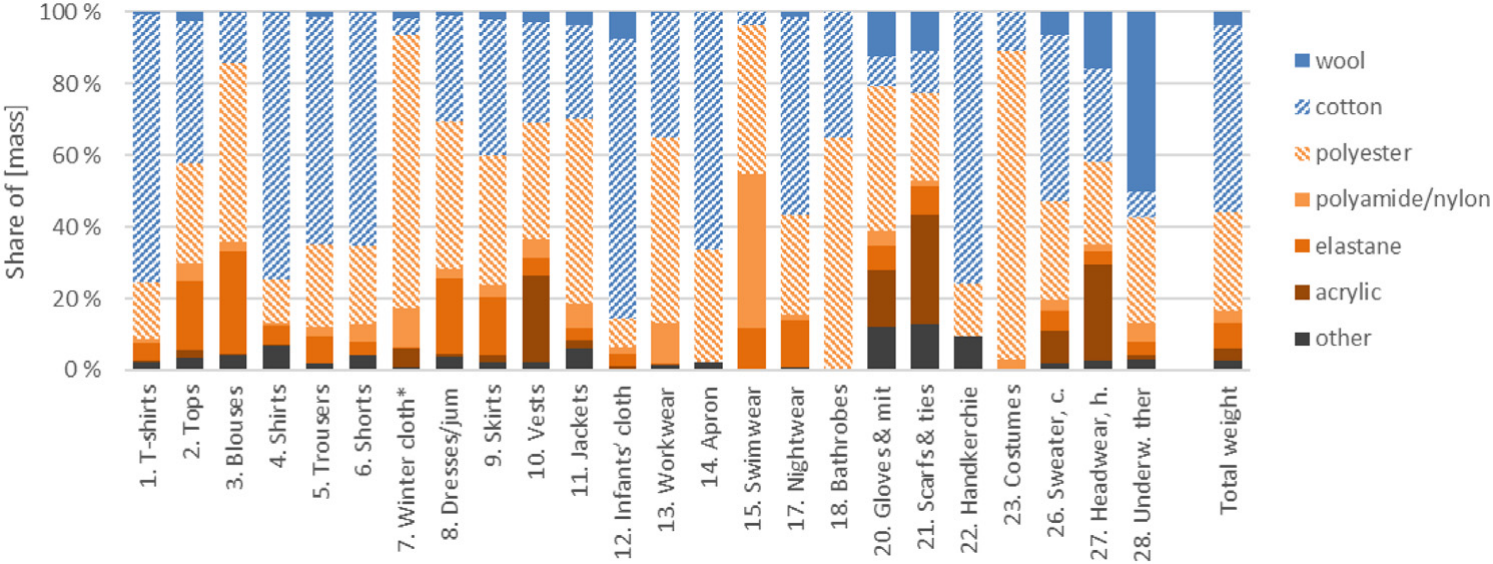
Material composition of garments categories and total mass across all samples, considering only single-layer garments. Complete category names provided in Table 2.

Of the full dataset, 13 955 items are single-layer garments. Fiber data is recognizable in the care label for 10 600 items, representing 64.4 % of samples. Fig. 4 shows the weight of main fibers in single-layer samples, with fiber material each <1 wt-% of total mass shown as “other”.

### 4.6 Imagery

Photography with Kodak Pixpro FZ55 using a standard tripod ring light. Limited guides were provided for the imagery, only that the images should be made on light background and show the entire garment and sample number.

## Limitations

The samples appear from collection partners and the available collection points and are not statistically representative of all Norwegian post-consumer textiles. Only urban areas are included. The sample period is June–August 2025, which will introduce seasonal bias. The study does not use randomization, stratified sampling, or probability sampling.

A standardized scale was not available at all locations, resulting in uncertainty in garment weight.

Lighting, distance, camera orientation, and background conditions for imagery were not standardized and therefore image-based machine learning applications should be undertaken with caution.

Some of the attributes require a level of subjective interpretation which leads to variation in the data, e.g., product categorization, color, manufacturing method and print type. Subjectivity also concerns grading of degree-of-wear and suitability for further use.

A standardized, unified and concise sampling and evaluation protocol would better support in-depth analysis of, among other things, fiber, geographic factors and longitudinal trends, and increase its value for policymakers, industry, and researchers. Data presented here forms a basis for development of such protocols.

## CRediT author statement

**Johan Berg Pettersen**: Conceptualization, Writing, Supervision, Project administration, Data curation. **Solveig Aarak**: Methodology, Writing, Supervision, Data collection, Data curation, Project administration. **Edyy Vanessa Peña Benítez**: Data collection, Data curation, Methodology, Writing. **Nazia Nourin Moury**: Data collection, Data curation, Methodology, Writing.

## Ethics statement

The authors confirm they have read and follow the ethical requirements for publication in Data in Brief and that the current work does not involve human subjects, animal experiments, or any data collected from social media platforms.

## Data Availability

ZenodoNorway post-consumer garment data and images: 16 464 clothing samples from donations and discards (Original data).

## Appendix Supplementary materials

Image, application 1
